# Supplementary material for: Predicting past and future SARS-CoV-2-related sick leave using discrete time Markov modelling
Source: PLoS One. 2022 Aug 12;17(8):e0273003. doi: 10.1371/journal.pone.0273003 (PMC9374214; doi:10.1371/journal.pone.0273003)
Supplement: S1 Text — (PDF) [file pone.0273003.s001.pdf]

Technical details

We used a discrete time Markov model to estimate the transition probabilities. Tutz and Schmid (2016) provides details on discrete time survival models, including the use of multinomial models to model for the one-step transition probabilities. Let $Y(t)$ be the state at time t for covariates $\boldsymbol{x}$, and define the transition probability $P_{ij}(s,t \mid \boldsymbol{x})=\Pr(Y(t)=j \mid Y(s)=i, \boldsymbol{x})$. In words, $P_{ij}(s,t \mid \boldsymbol{x})$ is the probability of being in state j at time t given being in state i at time s for covariates x. From the multinomial regression models, we have $P_{ij}(t,t+1 \mid \boldsymbol{x})$, with a vector $\boldsymbol{\beta}_{ij}$ of regression parameters for each sick leave state i in the preceding week to state j in the current week.

The multinomial regression models are defined as $P_{ij}(t,t+1|\boldsymbol{x}) = \frac{\exp(\boldsymbol{\beta}_{ij}^T\boldsymbol{x})}{\sum_k \exp(\boldsymbol{\beta}_{ik}^T\boldsymbol{x})}$, where $\boldsymbol{\beta}_{ii}=\mathbf{0}$. We can now define the matrix $\boldsymbol{P}(s,t|\boldsymbol{x}) = (P_{ij}(s,t|\boldsymbol{x}))$. Using Chapman-Kolmogorov equation, we have that $\boldsymbol{P}(s,t+1|\boldsymbol{x}) = \boldsymbol{P}(s,t|\boldsymbol{x})\boldsymbol{P}(t,t+1|\boldsymbol{x})$, so that we can construct the longer-term transition probability matrix as a product of one-step transition probability matrices.

We also standardized the transition probabilities over counterfactual covariate distributions. Let $\widehat{\boldsymbol{x}}_m$ be a counterfactual covariate vector with frequency w_m based on the person-time observed in the cohort. Then we can calculate the standardized transition probabilities by

$$\widehat{\boldsymbol{P}}(s,t) = \frac{\sum_m w_m \boldsymbol{P}(s,t|\widehat{\boldsymbol{x}}_m)}{\sum_m w_m}$$

For variance estimation of $P_{ij}(s,t|\boldsymbol{x})$, we can numerically calculate

$$\frac{\partial P_{ij}(s,t|\boldsymbol{x})}{\partial \beta_{ikm}} = \frac{P_{ij}(s,t|\boldsymbol{x})|_{\boldsymbol{\beta}_{ik}=\widehat{\boldsymbol{\beta}}_{ik}+\boldsymbol{e}_{ikm}\epsilon} - P_{ij}(s,t|\boldsymbol{x})|_{\boldsymbol{\beta}_{ik}=\widehat{\boldsymbol{\beta}}_{ik}-\boldsymbol{e}_{ikm}\epsilon}}{2\epsilon}$$

where ϵ is a small number (e.g. 10^{-5}) and $\boldsymbol{e}_{ikm}$ is a vector of zeroes of length $\boldsymbol{\beta}_{ik}$ except for index m, which takes value 1. If we stack the coefficients $\boldsymbol{\beta}$ and define the full covariance matrix $\boldsymbol{\Sigma}$ as being block-diagonal, then we can calculate the variance using the Delta method, such that

$$var\left(P_{ij}(s,t|\boldsymbol{x})\right) = \left(\frac{\partial P_{ij}(s,t|\boldsymbol{x})}{\partial \boldsymbol{\beta}}\right)^T \boldsymbol{\Sigma} \left(\frac{\partial P_{ij}(s,t|\boldsymbol{x})}{\partial \boldsymbol{\beta}}\right)$$

Confidence intervals were calculated using a logit transformation. We can similarly calculate the variance and confidence intervals for the standardized $\widehat{\boldsymbol{P}}(s,t)$. We further calculated point estimates, variances and confidence intervals for (a) the sum of the standardized transition probabilities for partial and full sick leave and (b) the log ratio of the standardized transition probabilities for the baseline covariate distribution compared with the referent period (where the estimates from the two intervals are independent), together with the attributable fraction for sick leave that could be attributed to COVID-19 infections. The variance and confidence interval calculations were also based on the delta method.
