# Supplementary material for: Predicting past and future SARS-CoV-2-related sick leave using discrete time Markov modelling
Source: PLoS One. 2022 Aug 12;17(8):e0273003. doi: 10.1371/journal.pone.0273003 (PMC9374214; doi:10.1371/journal.pone.0273003)

Figure S1 Percentages of sick leave for all employees in Karolinska University Hospital in year 2019 and 2020.

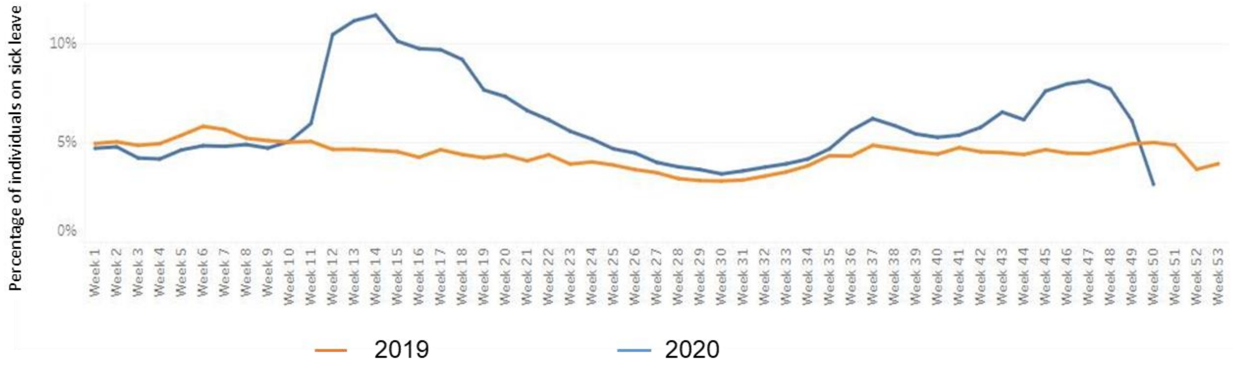

Supplement: S1 Fig — (PDF) [file pone.0273003.s002.pdf]
