# Supplementary material for: Predicting past and future SARS-CoV-2-related sick leave using discrete time Markov modelling
Source: PLoS One. 2022 Aug 12;17(8):e0273003. doi: 10.1371/journal.pone.0273003 (PMC9374214; doi:10.1371/journal.pone.0273003)
Supplement: S2 Fig — (PDF) [file pone.0273003.s003.pdf]

Figure S2 Predicted one-week transition probabilities from partial sick leave and full sick leave by calendar week.

A)

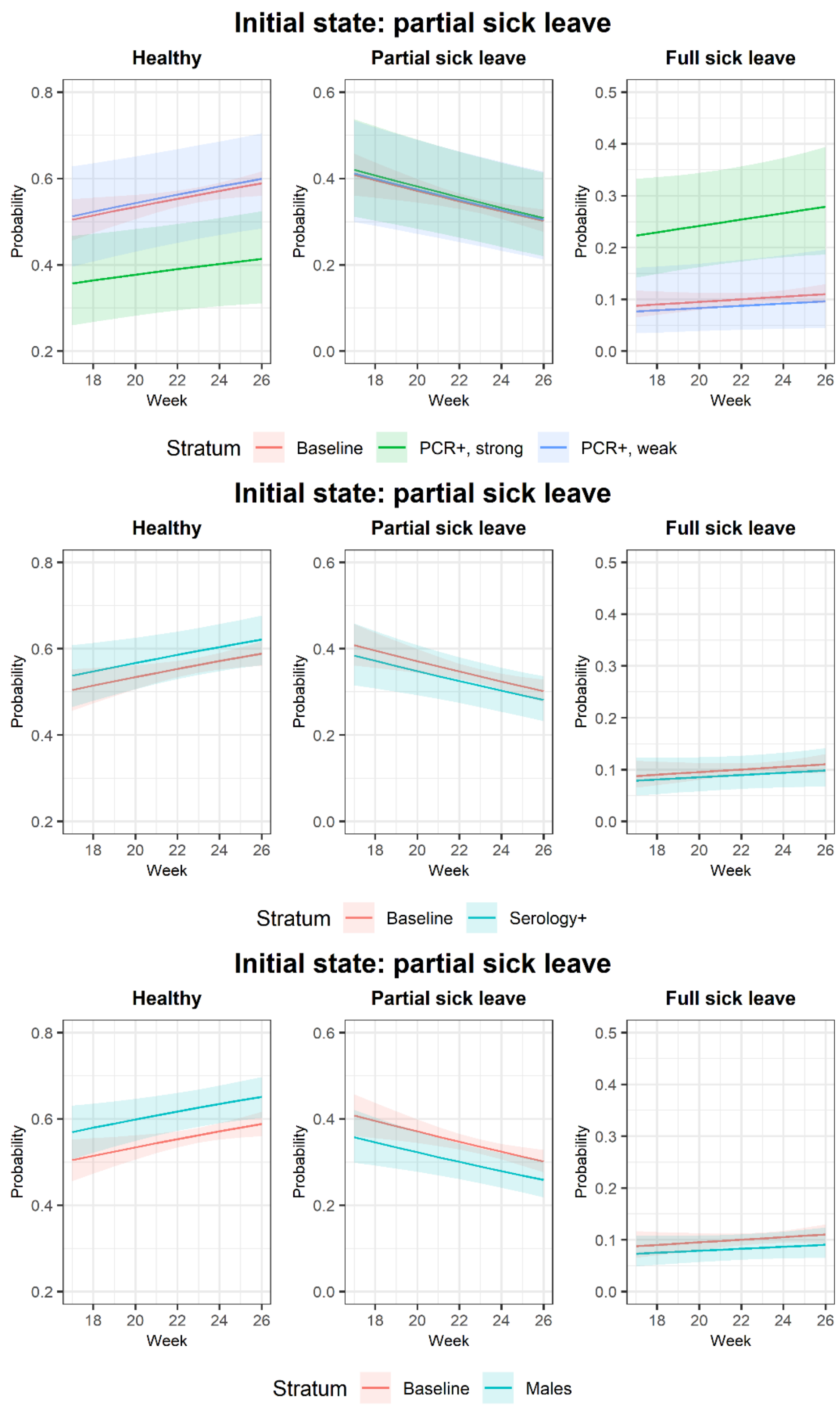

B)

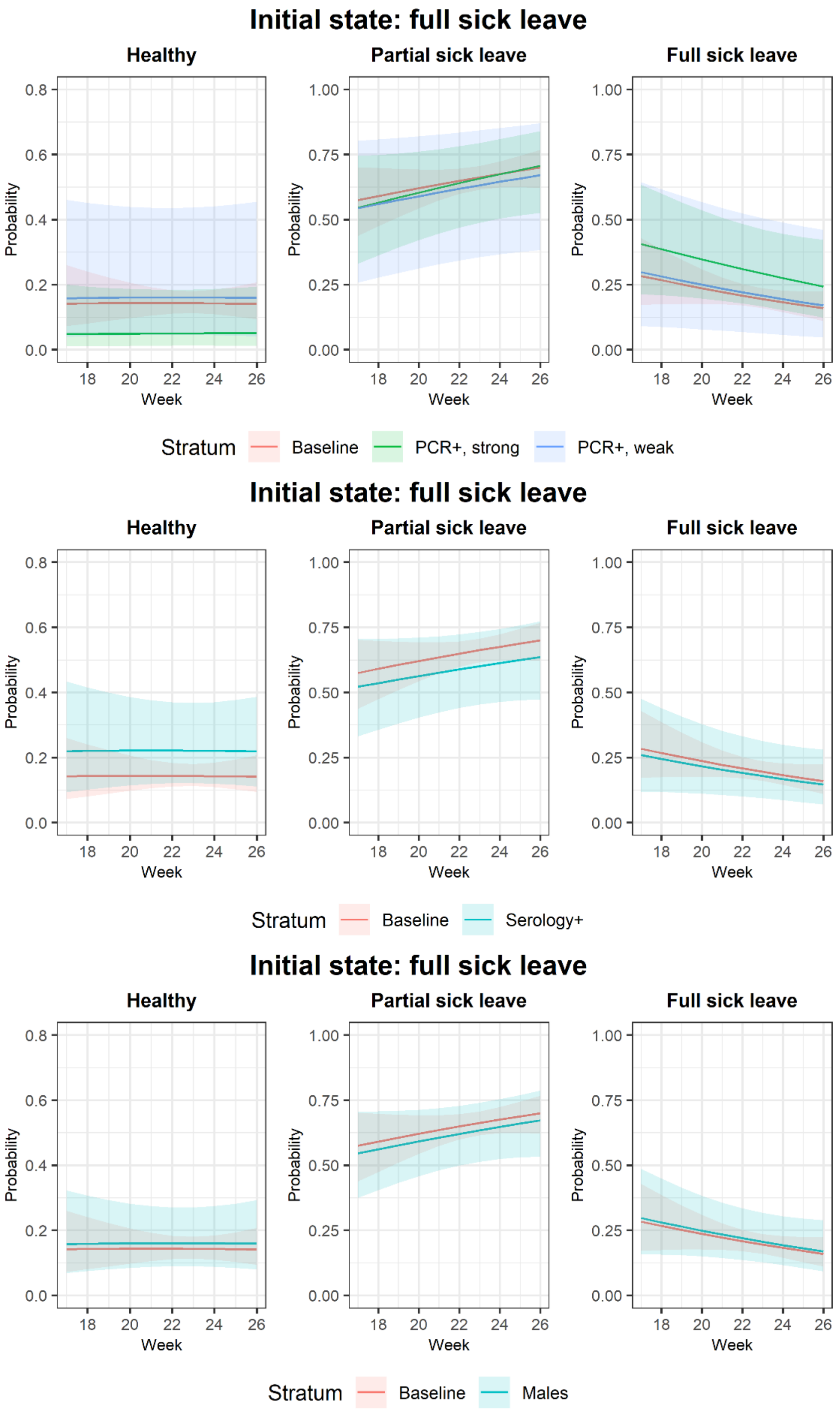

Initial state: full sick leave
Healthy
Partial sick leave
Full sick leave
Probability
Week
0.0
0.2
0.4
0.6
0.8
0.00
0.25
0.50
0.75
1.00
18
20
22
24
26
Stratum
Baseline
PCR+, strong
PCR+, weak
Initial state: full sick leave
Healthy
Partial sick leave
Full sick leave
Stratum
Baseline
Serology+
Initial state: full sick leave
Healthy
Partial sick leave
Full sick leave
Stratum
Baseline
Males
