# Supplementary material for: Predicting past and future SARS-CoV-2-related sick leave using discrete time Markov modelling
Source: PLoS One. 2022 Aug 12;17(8):e0273003. doi: 10.1371/journal.pone.0273003 (PMC9374214; doi:10.1371/journal.pone.0273003)

Figure S3 Transition probabilities at baseline and referent period by calendar week.

A) Week 17 to week 27

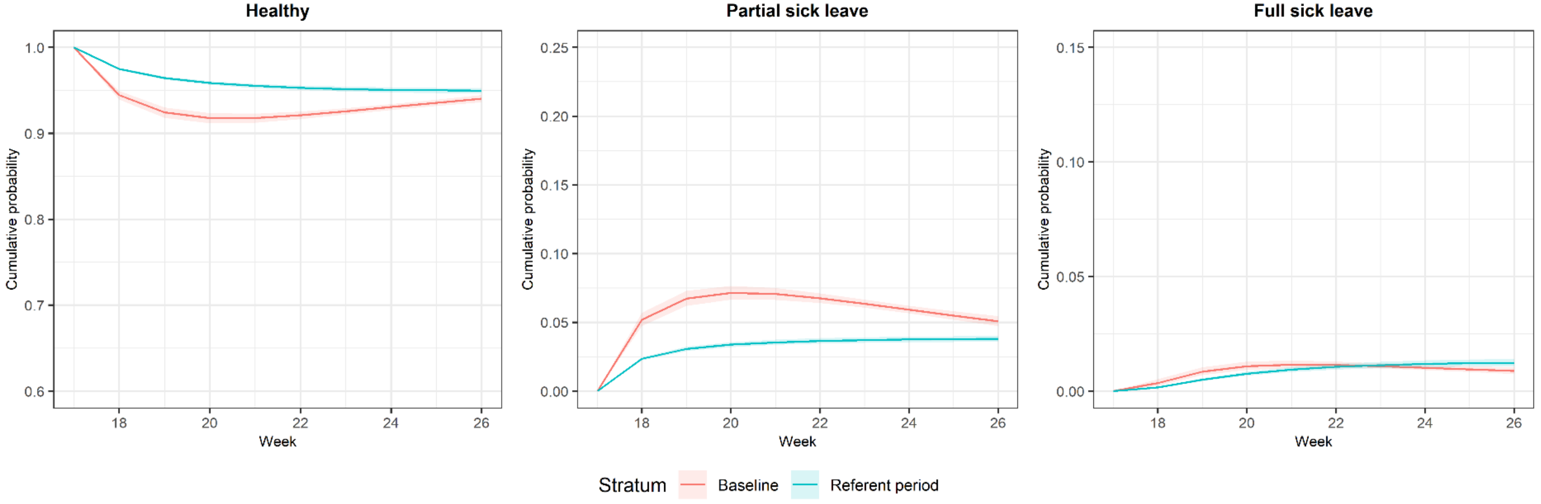


B) Week 17 to week 44

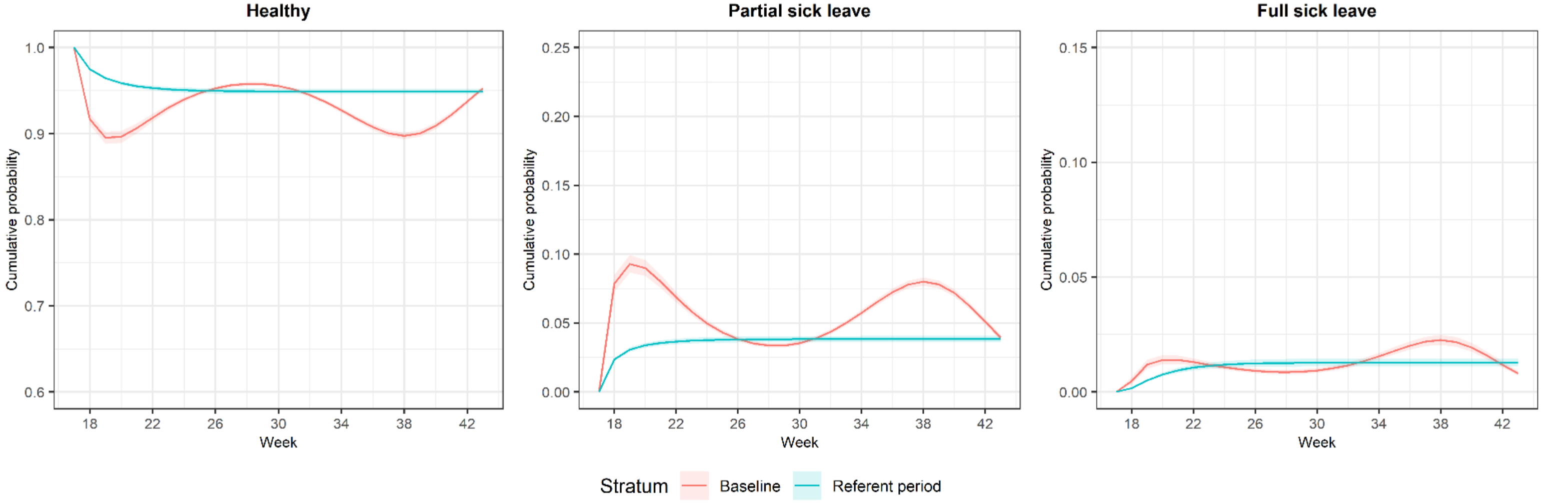

Supplement: S3 Fig — (PDF) [file pone.0273003.s004.pdf]
