# Supplementary material for: Predicting past and future SARS-CoV-2-related sick leave using discrete time Markov modelling
Source: PLoS One. 2022 Aug 12;17(8):e0273003. doi: 10.1371/journal.pone.0273003 (PMC9374214; doi:10.1371/journal.pone.0273003)

Figure S4 Predicted one-week transition probabilities from initial healthy state up to week 44 by calendar week

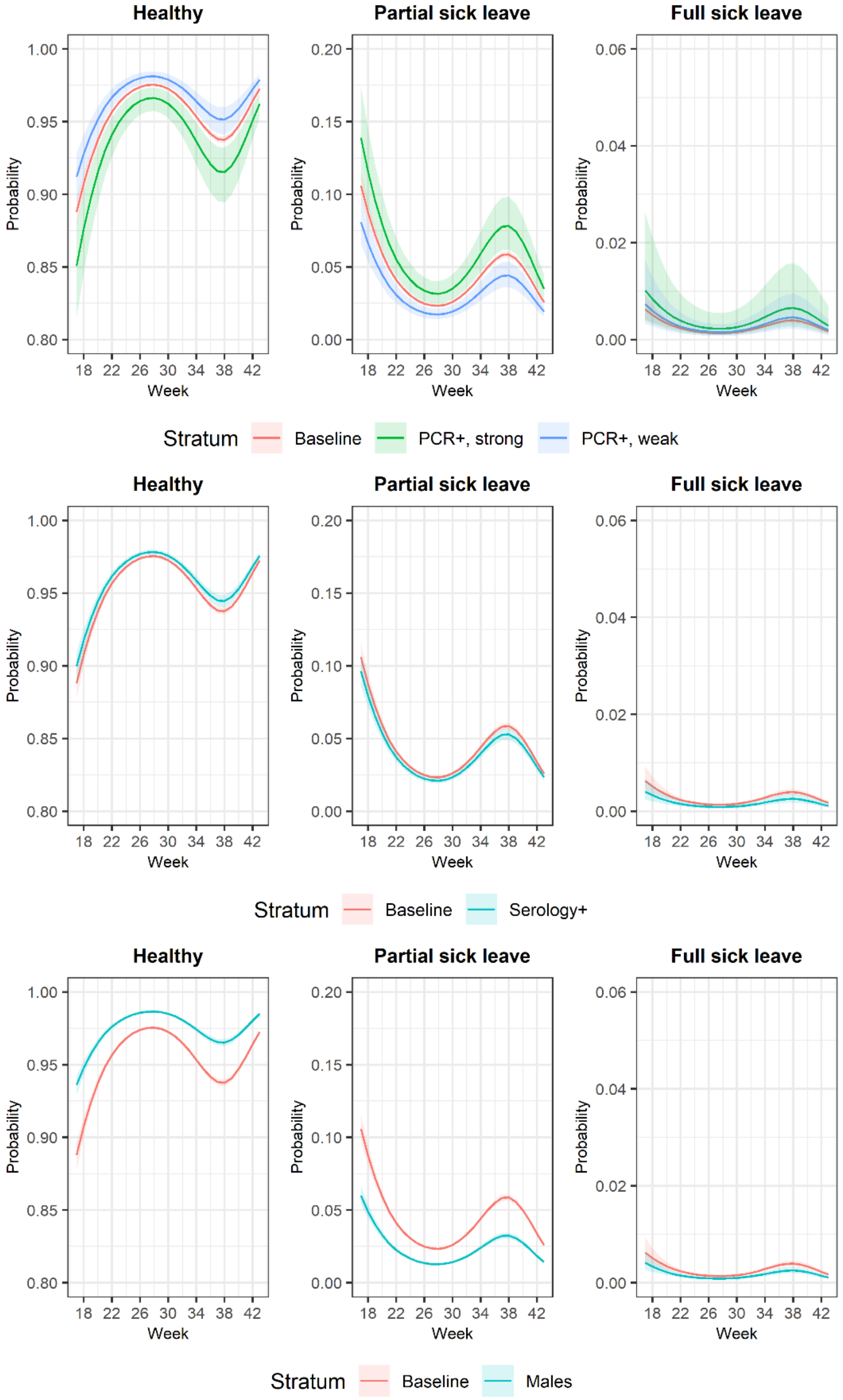

Supplement: S4 Fig — (PDF) [file pone.0273003.s005.pdf]
