# Supplementary figures and images for: Predicting past and future SARS-CoV-2-related sick leave using discrete time Markov modelling
Source: PLoS One. 2022 Aug 12;17(8):e0273003. doi: 10.1371/journal.pone.0273003 (PMC9374214; doi:10.1371/journal.pone.0273003)

Figure S5 Transition probabilities up to calendar week 44 by calendar week.

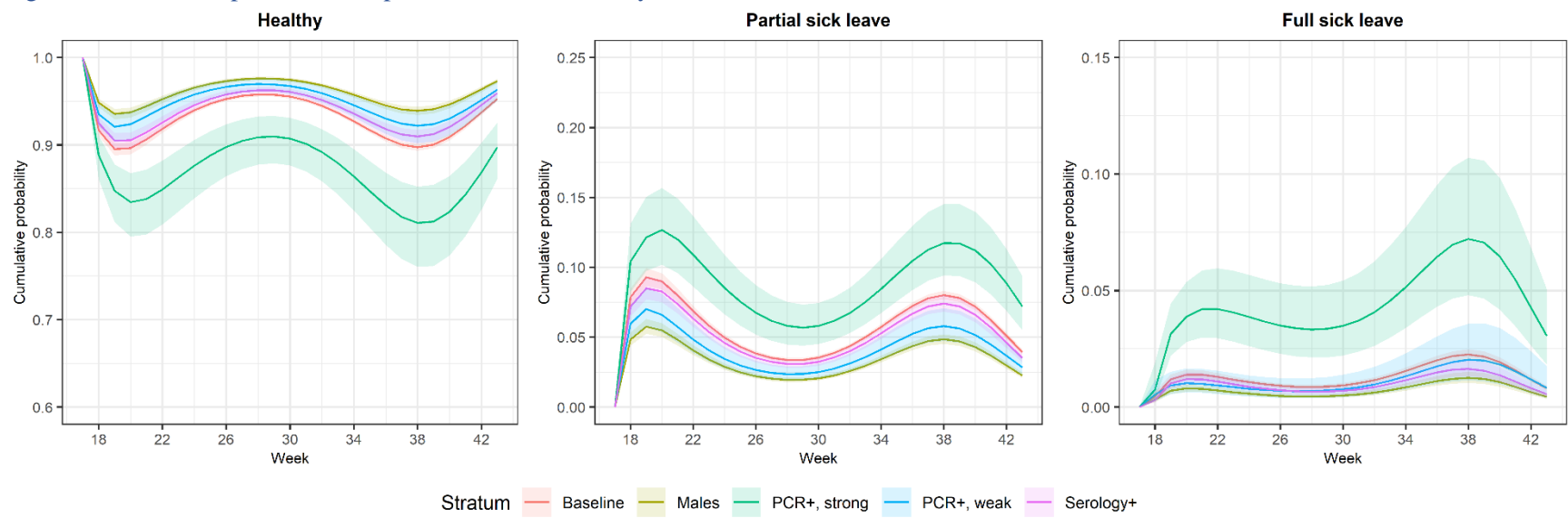

Supplement: S5 Fig — (PDF) [file pone.0273003.s006.pdf]
