# Supplementary material for: Predicting past and future SARS-CoV-2-related sick leave using discrete time Markov modelling
Source: PLoS One. 2022 Aug 12;17(8):e0273003. doi: 10.1371/journal.pone.0273003 (PMC9374214; doi:10.1371/journal.pone.0273003)
Supplement: S1 Table — (PDF) [file pone.0273003.s007.pdf]

Table S1 Frequency distribution of the transitions across sick leave state up to calendar week 27.

	Sick leave state at week n+1			
**Initial sick leave state at week n**	**Healthy**	**Partial sick leave**	**Full sick leave**	**Total**
**Healthy**	67 267	2 760	160	70 187
**Partial sick leave**	2 289	1 330	412	4 031
**Full sick leave**	81	359	114	554
**Total**	69 637	4 449	686	74 772
