# Supplementary material for: Predicting past and future SARS-CoV-2-related sick leave using discrete time Markov modelling
Source: PLoS One. 2022 Aug 12;17(8):e0273003. doi: 10.1371/journal.pone.0273003 (PMC9374214; doi:10.1371/journal.pone.0273003)
Supplement: S2 Table — (PDF) [file pone.0273003.s008.pdf]

Table S2 Odds ratios of sick leave state after the week of sampling up to calendar week 27, with additional adjustment of age and contact with patients.

a) Initial state: healthy

	**To sick leave**	
	**Partial sick leave**	**Full sick leave**
**Serological results**		
Positive vs. Negative	0.70 (0.60-0.82)	0.78 (0.41-1.49)
**PCR results**		
Strongly positive vs. Negative	1.58 (1.01-2.47)	2.98 (0.73-12.18)
Weakly positive vs. Negative	0.77 (0.52-1.13)	1.48 (0.45-4.88)
**Gender**		
Male vs Female	0.50 (0.44-0.57)	0.65 (0.40-1.06)
**Age groups**		
<30 vs 30-39	1.09 (0.95-1.24)	0.68 (0.35-1.31)
40-49 vs 30-39	0.76 (0.68-0.86)	0.82 (0.51-1.32)
50-59 vs 30-39	0.66 (0.58-0.74)	0.73 (0.44-1.20)
60+ vs 30-39	0.50 (0.43-0.58)	0.57 (0.30-1.07)
**Contact with patient**		
Yes vs. No	1.25 (1.13-1.38)	1.32 (0.87-2.01)

- Adjust for PCR, serology results, gender, age groups, contact with patients and calendar period with one degree of freedom.

b) Initial state: partial sick leave

	To sick leave	
	**Healthy**	**Full sick leave**
**Serological results**		
Positive vs. Negative	1.13 (0.88-1.46)	0.97 (0.63-1.49)
**PCR results**		
Strongly positive vs. Negative	0.67 (0.41-1.08)	2.50 (1.43-4.40)
Weakly positive vs. Negative	0.99 (0.62-1.61)	0.84 (0.36-1.99)
**Gender**		
Male vs Female	1.27 (1.02-1.57)	0.95 (0.66-1.36)
**Age groups**		
<30 vs 30-39	1.19 (0.95-1.49)	1.01 (0.70-1.45)
40-49 vs 30-39	0.98 (0.81-1.18)	0.77 (0.57-1.06)
50-59 vs 30-39	0.92 (0.76-1.11)	0.92 (0.68-1.25)
60+ vs 30-39	1.06 (0.82-1.36)	1.37 (0.94-1.99)
**Contact with patients**		
Yes vs. No	0.87 (0.74-1.02)	0.97 (0.75-1.27)

- Adjust for PCR, serology results, gender, age groups, contact with patients and calendar period with one degree of freedom.

c) Initial state: full sick leave

	To sick leave	
	**Healthy**	**Partial sick leave**
**Serological results**		
Positive vs. Negative	1.38 (0.52-3.69)	0.90 (0.40-1.99)
**PCR results**		
Strongly positive vs. Negative	0.22 (0.05-1.04)	0.61 (0.28-1.34)
Weakly positive vs. Negative	1.58 (0.27-9.35)	1.06 (0.25-4.53)
**Gender**		
Male vs Female	1.09 (0.47-2.55)	0.87 (0.47-1.64)
**Age groups**		
<30 vs 30-39	1.70 (0.70-4.11)	1.02 (0.53-1.97)
40-49 vs 30-39	3.32 (1.41-7.81)	2.27 (1.16-4.46)
50-59 vs 30-39	1.81 (0.78-4.20)	1.95 (1.06-3.60)
60+ vs 30-39	1.10 (0.38-3.14)	1.32 (0.65-2.67)
**Contact with patients**		
Yes vs. No	1.43 (0.66-3.10)	0.98 (0.57-1.67)

- Adjust for PCR, serology results, gender, age groups, contact with patients and calendar period with one degree of freedom.
