# Supplementary material for: Predicting past and future SARS-CoV-2-related sick leave using discrete time Markov modelling
Source: PLoS One. 2022 Aug 12;17(8):e0273003. doi: 10.1371/journal.pone.0273003 (PMC9374214; doi:10.1371/journal.pone.0273003)
Supplement: S3 Table — (PDF) [file pone.0273003.s009.pdf]

Table S3 Frequency distribution of the transitions across sick leave states up to week 54.

	Sick leave state at week n+1			
**Initial sick leave state at week n**	**Healthy**	**Partial sick leave**	**Full sick leave**	**Total**
**Healthy**	239 227	8 156	517	247 900
**Partial sick leave**	8 211	4 161	1 208	13 580
**Full sick leave**	459	1 266	1 367	3 092
**Total**	247 897	13 583	3 092	264 572
