# Supplementary material for: Predicting past and future SARS-CoV-2-related sick leave using discrete time Markov modelling
Source: PLoS One. 2022 Aug 12;17(8):e0273003. doi: 10.1371/journal.pone.0273003 (PMC9374214; doi:10.1371/journal.pone.0273003)
Supplement: S4 Table — (PDF) [file pone.0273003.s010.pdf]

Table S4 Odds ratios of sick leave after the week of sampling up to calendar week 54.

a) Initial state: healthy

	**To sick leave**	
	**Partial sick leave**	**Full sick leave**
**Serological results**		
Positive vs. Negative	0.90 (0.83-0.97)	0.64 (0.45-0.91)
**PCR results**		
Strongly positive vs. Negative	1.37 (1.07-1.76)	1.70 (0.70-4.12)
Weakly positive vs. Negative	0.74 (0.60-0.91)	1.14 (0.56-2.35)
**Gender**		
Male vs Female	0.54 (0.50-0.57)	0.62 (0.48-0.80)

- Adjust for PCR, serology results, gender and calendar period with one degree of freedom.

b) Initial state: partial sick leave

	**To sick leave**	
	**Healthy**	**Full sick leave**
**Serological results**		
Positive vs. Negative	0.97 (0.85-1.10)	1.06 (0.85-1.32)
**PCR results**		
Strongly positive vs. Negative	0.77 (0.55-1.09)	2.50 (1.65-3.77)
Weakly positive vs. Negative	1.25 (0.90-1.72)	0.76 (0.41-1.42)
**Gender**		
Male vs Female	1.15 (1.03-1.29)	0.89 (0.73-1.10)

- Adjust for PCR, serology results, gender and calendar period with one degree of freedom.

c) Initial state: full sick leave

	**To sick leave**	
	**Healthy**	**Partial sick leave**
**Serological results**		
Positive vs. Negative	1.76 (1.19-2.60)	1.56 (1.15-2.12)
**PCR results**		
Strongly positive vs. Negative	0.50 (0.22-1.14)	0.97 (0.59-1.58)
Weakly positive vs. Negative	0.61 (0.23-1.58)	0.61 (0.30-1.22)
**Gender**		
Male vs Female	1.37 (1.00-1.89)	0.97 (0.75-1.26)

- Adjust for PCR, serology results, gender and calendar period with one degree of freedom.
